# Peer-Peer Cultural Value Mismatch in the Dormitory During the Transition to College: Antecedents and Correlates

**DOI:** 10.3726/jicir.2022.1.0004

**Published:** 2023

**Authors:** Yolanda Vasquez-Salgado, Patricia M. Greenfield, Shu-Sha Angie Guan, Lucy Gonzalez, Darby A. Tarlow

**Affiliations:** California State University, Northridge; University of California, Los Angeles; California State University, Northridge; California State University, Northridge; University of California, Los Angeles

**Keywords:** first-generation college students, cultural value mismatch, cultural mismatch, peer relations, dormitory roommates, transition to college, academic outcomes

## Abstract

This research focuses on *peer-peer cultural value mismatch* – perceived mismatch between collectivistic ideologies and practices of one student and individualistic ideologies and practices of another – among students living in the dormitories during the transition to college. Two survey studies examined the antecedents and correlates of two types of mismatch: (1) reciprocation mismatch: giving or offering a material or service to one’s roommate but not receiving anything in return; and (2) not thinking of the other: feeling as though roommates are not considerate of one’s feelings or schedule. Study 1: A sample of 110 students in their first year of college showed that being a first-generation college student increased the likelihood of experiencing reciprocation mismatch. Both forms of mismatch predicted experiences of psychological distress, reports of academic problems, and lower grades. Study 2: A sample of 152 (76 dormitory roommate pairs) first-year college students revealed that social-class differences in parental education between dormitory roommates predicted students’ experiences with reciprocation mismatch. Students of lower parental education than their roommate reported significantly more mismatch. More mismatch experience was in turn linked to significantly higher levels of academic problems during the transition to college. Implications for research, residential life, and intervention are discussed.

## Introduction

I volunteer to do things for you because I understand we all need that helping hand…but then when I don’t get it back its just like…we had math and we’re both taking [it]…she had one resource that I needed and she heard me…struggling for it and she didn’t do anything about it… (Latinx first-generation college student; Burgos-Cienfuegos et al., 2015)^1^

The quote above represents the qualitative, lived experience, of a first-generation college student – one whose parents had no more than a high-school education. This student supported her roommate, but felt as though she did not receive support back in return. This experience of reciprocation mismatch is part of a broader phenomenon known as *peer-peer cultural value mismatch* – perceived mismatch between collectivistic ideologies and practices of one student and individualistic ideologies and practices of another. Collectivistic ideologies prioritize group goals and community cohesion; individualistic ideologies prioritize one’s personal needs and goals (Greenfield, 2009). Mismatch between collectivistic and individualistic peer behavior can be a source of stress for first-generation college students during the transition to college (Burgos-Cienfuegos et al., 2015).

The purpose of the current research is to extend our knowledge base by quantitatively examining the antecedents and correlates of peer-peer cultural value mismatch. Our findings on this topic have social importance, given the ever growing diversity in higher education and society as a whole.

## Rationale for an Empirical Investigation of Dormitory Roommates

Decades of research have demonstrated the powerful impact, both positive and negative, that peers may have on students’ development (Dennis et al., 2005; Juang et al., 2016). This impact begins in adolescence, once youth begin to spend more time with their peers; it continues as they move on to college (Lightfoot et al., 2018), at which point a significant percentage of students live in campus dormitories and have roommates (American College Health Association, 2019). At the University of California, Los Angeles (UCLA), where the present research was conducted, almost all students live in the dormitories in their first year of college. Thus, in investigating peer relationships, empirical work among roommates living in dormitories is absolutely necessary.

Roommate relationships may be particularly important during the transition to college. For the very first time, many students move away from their family home and live with someone that is a nonfamily member. Nearly 50 % of first-year college students in the U.S. report “frequent” or “occasional” conflicts with roommates (Liu et al., 2008). Conflicts with roommates may be particularly impactful because they live in close proximity to one another, and are somewhat permanent peers, at least for a quarter/semester or an academic year. Indeed, roommate conflict negatively impacts students’ health and academic adjustment (e.g., stress, academic grades; Erb et al., 2014). However, only one study has described intergroup conflicts or situations that might occur in the dormitory (Jaggers & Iversen, 2012; i.e., racial stereotypes and interracial tension). Nonetheless, even this study did not deal with the issue of differing cultural values, the subject of our research. Though there is some work documenting that ethnic similarity (Bresnahan et al., 2009) and similar communication styles (Martin & Anderson, 1995) promote positive interpersonal outcomes among roommates, we have found no work on the role of social class and cultural value differences between roommates. Our research is a first attempt to fill these gaps by investigating peer-peer cultural value mismatch and the role of social class differences among roommates in experiencing this kind of mismatch.

## Previous Research on Peer-Peer Cultural Value Mismatch

The concept of peer-peer cultural value mismatch (previously termed, peer-peer value conflicts) in a post-secondary education setting, was first introduced via a qualitative study with Latinx first-generation college students (71 % female; parental education range: no formal education – graduated from high school) who were in their first year of study at UCLA, the site of the present two studies (Burgos-Cienfuegos et al., 2015). Students took part in a group interview that ranged from 3 to 7 students. In the group interview, 57 % of these Latinx first-generation college students reported having experienced a peer-peer cultural value mismatch situation in which their behavior was collectivistic, but their peer’s behavior was individualistic; almost always these experiences were with a roommate.

The experiences were of two different types: (1) *Reciprocation mismatch*: they gave or offered a material or a service to their roommate but did not receive anything in return; or (2) *Not thinking of the other*: students’ roommates were not considerate of their feelings or schedule (Burgos-Cienfuegos et al., 2015). There were indications that Latinx first-generation college students felt these situations played a negative role in their college adjustment, particularly in regard to stress and negative emotions (components of mental health), as well as in the ability to control their attention (an aspect of mental health and academics). Though students denied that such situations impacted their grades, this response may have been due to the fact that they felt “family” had a stronger impact on them (Vasquez-Salgado et al., 2015). In other words, students felt that cultural mismatch between family and academics had a stronger impact on their grades than did cultural mismatch situations with peers. Nonetheless, poor peer relations can undermine students’ academic adjustment (Erb et al., 2014).

This qualitative study was foundational to the current research in several ways. First, it served as the basis for developing a novel instrument of peer-peer cultural value mismatch. Second, the qualitative results inspired our interest in quantitatively examining antecedents and correlates of this mismatch. We were particularly interested in examining the relation between peer-peer-cultural value mismatch and academic adjustment. Though students did not perceive that these peer-peer situations impacted their academics, we sought to test the implications in an objective, quantitative fashion – given that prior research suggested consequences. Third, recognizing that this study was conducted with one group – Latinx first-generation college students, we aimed to expand the diversity of our samples. Greater diversity would ensure that our findings would be more generalizable to students from other backgrounds, particularly, first-generation college students from diverse ethnic groups.

## Rationale for an Emphasis on First-Generation College Students

Nearly one-third of first-time freshmen across the nation are first-generation college students (U.S. Department of Education, 2018). In some studies, they are defined as students whose parents had no postsecondary education (e.g., Toutkoushian et al., 2019); in other studies, they are defined as students whose parents have not attained a four-year degree (e.g., Stephens et al., 2012a). However they are defined, they are more likely to drop out of college or take longer than their continuing-generation college peers (students whose parents had contrasting levels of postsecondary education) to earn their degrees (U.S. Department of Education, 2018; DeAngelo et al., 2011). These educational disparities have led researchers to investigate contributing factors. Peer relations have emerged as an important factor, especially for first-generation students. Poor peer relations contribute to negative adjustment outcomes for all college students (Gan et al., 2019; Juang et al., 2016; Maunder, 2018; Sadoughi & Hesampour, 2016). However, the contribution is greater for first-generation college students (Dennis et al., 2005; Jenkins et al., 2013; Posselt & Lipson, 2016), including those residing at college (Sriram et al., 2020).

This impact may stem from the fact that first-generation college students represent a minority group within university settings. For this reason, their collectivistic, cultural practices, such as those involving the importance of community (Stephens et al., 2012a), are not always aligned with or reciprocated by their majority peers (Burgos-Cienfuegos et al., 2015). Therefore, first-generation college students are at the forefront of our investigation because this is the population at highest risk of experiencing cultural value mismatch.

## Theoretical Framework

Two seminal theories guided the current research. The first, Theory of Social Change, Culture, and Human Development, describes the antecedents of cultural mismatch at a broad scale (Greenfield, 2009). The second, Cultural Mismatch Theory, is a focused theory that describes the antecedents and consequences of a general notion of cultural mismatch in a university setting, with first-generation college status being central in the experience of cultural mismatch (Stephens et al., 2012a; 2012b).

### Theory of Social Change, Culture, and Human Development

This theory conceptualizes cultural values as an adaptation to particular sociodemographic ecologies; it posits that movement from one ecology to another can result in a sense of cultural value mismatch or conflict (Greenfield, 2009). According to the theory, collectivistic values are adapted to ecologies in which formal education is limited and material resources are low – an ecology experienced by the parents of first-generation college students. In contrast, individualistic values are adapted to ecologies in which opportunity for formal education is great and material resources are more abundant – the ecology experienced by the parents of continuing generation college students, and the college environment itself. This theory predicts that, when individuals transition from an ecology characterized by collectivistic values to one that is more individualistic, cultural value mismatch can occur. Thus, central to the current research, this theory predicts that students with parents who have low levels of education will experience cultural value mismatch during their transition to post-secondary education. Empirical support for this theory has been gathered from research on Latinx youth from economically challenged homes at various stages in educational development (elementary: Trumbull et al., 2001; Greenfield & Quiroz, 2013; high school: C. Suárez-Orozco & M. Suárez-Orozco, 1995; four-year universities: Burgos-Cienfuegos et al., 2015; Vasquez-Salgado et al., 2015).

### Cultural Mismatch Theory

This theory provides a detailed account of cultural mismatch within four-year university settings (Stephens et al., 2012a). According to Cultural Mismatch Theory, four-year universities normalize independent or individualistic values. That is, priority is given to personal needs and goals. These values align with those of continuing-generation college students, but mismatch with the interdependent or collectivistic values that first-generation college students are socialized with at home. These students prioritize group goals and community cohesion. This situation results in cultural mismatch with the university culture for first-generation college students, but not for continuing-generation college students. This general sense of mismatch has been rigorously examined at research-centered institutions (private and public); it has been documented to cause a disruption in both health and academic performance during the first-year of college (Stephens et al., 2012a; Stephens et al., 2012b).

The health effect manifests in an increase in the stress hormone, cortisol, and negative emotions when a student is exposed to experimentally induced cultural mismatch. For first-generation college students, priming individualistic values produced a significantly larger stress response than priming collectivistic values (Stephens et al., 2012b). However, for continuing-generation college students, there was no significant difference in stress in the different value conditions. A similar pattern unfolded for academic-related tasks: poorer performance on such tasks for first-generation college students primed with individualistic values compared with collectivistic primes; no performance differences with different value primes for continuing generation students (Stephens et al., 2012a). Thus, this theory has led to empirical support for the existence of cultural value mismatch in a university setting and has shown its explanatory power. Central to the current research, it implies that first-generation college status is an antecedent to experiencing cultural value mismatch and that mismatch has negative consequences for health and academic adjustment during the transition to college.

### Theoretical Conceptualization of Peer-Peer Cultural Value Mismatch in the Dormitory

Based on these theoretical perspectives, we expected that the cultural orientation of first-generation college students (or students with lower levels of parental education) living in a dormitory would be more collectivistic, whereas the cultural orientation of continuing-generation roommates (or roommates with higher levels of parental education) would be more individualistic. The situation of social class mismatch among roommates thus sets the stage for peer-peer cultural value mismatch – the experience of mismatch between the more collectivistic ideologies and practices of one student and the more individualistic ideologies and practices of another (Burgos-Cienfuegos et al., 2015).

## Current Research

The current research advances prior theoretical frameworks by examining the antecedents and correlates of an understudied form of mismatch – peer-peer cultural value mismatch. In this research, we focus on the experience of peer-peer cultural value mismatch from the perspective of a student with a more collectivistic orientation living with a roommate who is more individualistic, as prior theory and research with students at four-year universities have indicated that cultural mismatch has greater consequences for first-generation than for continuing-generation college students (Stephens et al., 2012a; Stephens et al., 2012b; Vasquez-Salgado et al., 2021). In two quantitative studies we examined antecedents and correlates of peer-peer cultural value mismatch in multiethnic samples.

We created a novel measurement of peer-peer cultural value mismatch that was distributed to first-year students living in dormitories during the transition to college. The first study surveyed first-year dormitory residents without considering the sociodemographic characteristics of their roommates. The second study filled this gap by sampling roommate pairs. This sampling strategy allowed us to explore how social class differences between roommates relate to experiences with peer-peer cultural value mismatch.

The contribution of this research is to enhance understanding of cultural mismatch within a key social context for entering college students, the university dormitory. Extant research has largely focused on a general sense of cultural mismatch between students’ collectivistic values and the individualistic values of the university institution (Stephens et al., 2012a; Stephens et al., 2012b; Phillips et al., 2020). With the exception of our qualitative study (Burgos-Cienfuegos et al., 2015), the social context of peer-peer cultural value mismatch among dormitory roommates has not been explored. Our two quantitative studies begin to fill this gap.

## Hypotheses

1. In terms of antecedents, peer-peer cultural value mismatch is most likely to be experienced by first-generation college students (Study 1) or students whose parents have lower levels of education than their roommate’s parents (Study 2).
2. In terms of correlates, peer-peer cultural value mismatch is expected to have negative repercussions for health and academic development (Studies 1 and 2).

### Study 1

In Study 1, we sought to test the generality of our prior qualitative findings with Latinx first-generation college students in a sample of first-generation college students from a variety of ethnic backgrounds. We created a survey instrument to assess peer-peer cultural value mismatch experiences and distributed it to first-year students from diverse backgrounds living in university dormitories. Following up on our prior qualitative research, we focused on documenting quantitatively the sociodemographic antecedents, as well as the psychological and behavioral correlates of peer-peer cultural value mismatch.

## Hypotheses

### Antecedents of Peer-Peer Cultural Value Mismatch (Hypothesis 1)

The original study documented peer-peer cultural value mismatch in a sample of Latinx first-generation college students (Burgos-Cienfuegos et al., 2015). Latinx students are considered a disadvantaged minority group, as are Black students (Townsend et al., 2019). Thus, because we had a diverse sample, we incorporated disadvantaged minority background as a variable within our analysis of antecedents. Including this variable within our analyses is important as empirical research suggests that disadvantaged minority students (e.g., Latinx, Black) hold strong collectivistic values (e.g., Brannon et al., 2015; Fuligni & Pedersen, 2002; Greenfield et al., 2003); and engagement in collectivistic practices, such as forming communities, is important in fostering positive college experiences in this demographic (Boettcher et al., 2019). Thus, we needed to ensure that our findings regarding the role of parent education in this particular type of mismatch hold above and beyond one particular minority group.

Based on the work of Stephens and colleagues (2012a; 2012b), we hypothesized that first-generation college students would experience peer-peer cultural value mismatch more than their continuing-generation peers and that this relation would hold even after controlling for minority background. Such results would enable us to generalize the experience of peer-peer cultural value mismatch to first-generation college students from all ethnic backgrounds. We expected these relations to hold across two kinds of peer-peer cultural mismatch, one focused on reciprocation mismatch with one’s roommate, and the other focused on experiences with a roommate who does not think of the other person. Thus, a secondary goal of this hypothesis was to examine whether both types of mismatch reliably differentiate the experience of first-generation from that of continuing-generation students.

### Correlates of Peer-Peer Cultural Value Mismatch (Hypothesis 2)

Our hypothesis concerning the associated correlates that unfold with the experience of peer-peer cultural value mismatch are based on the general, theoretical notion that cultural mismatch negatively impacts health (Stephens et al., 2012b) and academic adjustment (Stephens et al., 2012a) during the transition to college. In addition, the specific, interconnected paths involving health and academic performance are based on the findings from our qualitative study of peer-peer cultural value mismatch among Latinx students as well as other literature.

**Figure 1.**
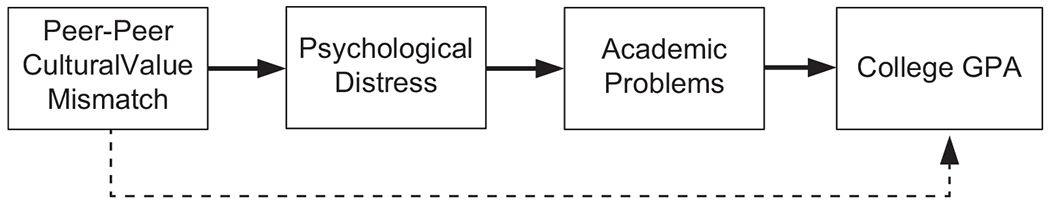
Hypothesized model for correlates of peer-peer cultural value mismatch. A dashed line indicates a non-significant relation was expected. Two separate models were tested, one of reciprocation mismatch and another for not thinking of the other.

In Burgos-Cienfuegos et al. (2015), Latinx first-generation college students reported that cultural value mismatch with their dormitory roommates made them feel distressed. This finding led to the first hypothesized link in our model of correlates: peer-peer cultural value mismatch will predict psychological distress (Figure 1). Moreover, correlational and experimental studies suggest that individuals who experience psychological distress are also prone to experiencing problems with regulating their attention (Liston et al., 2008; Mathews & MacLeod, 1985; Meyers et al., 2014; Yiend, 2010) and engagement in learning activities (Ramirez & Beilock, 2011; Rozek et al., 2019). These findings led to the second link in our correlates model: psychological distress, will, in turn, relate to higher levels of attention and learning problems, which we term, academic problems. Indeed, issues with regulating one’s attention furnished one topic of conversation among participants who experienced these peer-peer mismatch situations in the small groups studied by Burgos-Cienfuegos and colleagues (2015). A logical correlate of academic problems is lower grade point averages (GPAs). Our model therefore predicted a significant link from academic problems to GPA. Lastly, because we expected psychological distress and academic problems to serve as the main mechanisms that relate peer-peer cultural value mismatch to GPA, we did not expect a direct link in the model from mismatch to GPA. Instead, we predicted that peer-peer cultural value mismatch would have a significant indirect effect on GPA through the paths of the intervening variables (Figure 1). We expected these relations to hold in two separate models, one for reciprocation mismatch and one for not thinking of the other.

## Method

### Participants.

During the Spring quarter of their first year of college at UCLA, students were recruited via the psychology subject pool and flyers posted throughout campus. The only requirement was that participants had to be in their first year at UCLA. They could be transfer students, and there were five transfer students in the final sample. The average age of our final sample was 18.68 (*SD* = .90); the age range was 18 to 23 years (76 % were female).

A total of 137 students completed the survey. However, since our study sought to examine experiences with peer-peer cultural value mismatch in the dormitory, the sample size further decreased to 111 because we omitted students who did not live in the dorms. Finally, our sample size further decreased to 110 because one student did not complete the entire survey.

Thirty percent of the students in our sample were first-generation college students, meaning that neither parent held any experience with postsecondary education (please see further details in our measures section). Our sample was ethnically diverse (Asian = 35; Latinx = 30; White or European American = 32; Black or African American = 4; Multiracial or Other Ethnicities = 9). These percentages roughly corresponded to the distribution of ethnicities at UCLA (UCLA Undergraduate Admissions, 2018–2019).

## Measures

### Disadvantaged minority background.

Following Townsend et al. (2019), we distinguished between ethnic groups who are academically disadvantaged and those who are not. We labeled all students who identified themselves as Latinx, Black or African American or with one of these ethnic backgrounds and another background as 1 (disadvantaged minority). Students who identified with another ethnic background were labeled as 0 (e.g., White or European American, Asian); they were considered academically advantaged. This categorization aligns with definitions used by Townsend et al. (2019). It is important to note that by academically disadvantaged we are referring to the historical marginalization or minoritization of these ethnic groups in education and society (Vasquez-Salgado et al., 2023).

### First-generation college student status.

Students who were considered first-generation college students came from households where their parents had no form of postsecondary education (coded as 1). Students who reported that their parents had at least some postsecondary education or higher were labeled as continuing generation college students (coded as 0). This cut-point has been frequently used in the field (Demetriou et al., 2017; Saenz et al., 2007; Toutkoushian et al., 2019) and had particular ecological relevance to our population. Specifically, among the Latinx first generation college students at UCLA that took part in our prior qualitative study on peer-peer cultural value mismatch, the highest level of parental education was high school; thus, no parent had any form of postsecondary education.

### Peer-peer cultural value mismatch.

A 10-item measure assessing mismatch between the collectivistic ideologies and practices of dormitory residents and the individualistic ideologies and practices of their roommates was created using phenomena identified by Burgos-Cienfuegos et al. (2015). The measure assessed the frequency of two different types of peer-peer mismatch. The first type, reciprocation mismatch, included five-items; sample items included: “When going somewhere (e.g., store, coffee shop), I ask my roommate if he or she wants or needs anything but he or she never does the same for me,” and “I have shown support to my roommate when needed but he or she has not reciprocated support when I need it.”

The second type, not thinking of the other, included 5-items; sample items included: “I often find myself cleaning common areas (e.g., restroom, trash) that my roommate and I both use because he or she never helps clean,” and “My roommate makes a lot of noise (e.g., watches television, talks on the phone) when I am trying to study.” All items were prefaced with the following statement: “Since you started rooming with X, please state how often you have experienced the following with him or her…”. Responses ranged from 1 (*Never*) to 4 (*Frequently*). The Cronbach alphas were .89 and .78, respectively, indicating acceptable internal consistency among the items (Newton & Rudestam, 1999).

A principal components analysis with promax rotation and Kaiser normalization was conducted with the items of the peer-peer cultural value mismatch measure. The analysis resulted in a Kaiser-Meyer-Olkin (KMO) coefficient of .818 (well above the recommended .50) and a Bartlett’s Test of Sphericity of *χ*^2^(45) = 556.07, *p* < .001, suggesting significant sampling adequacy. As shown in Table 1, the analysis yielded two factors with Eigenvalues greater than 1.00 and item loadings of .40 or higher. As expected, the ten items of the peer-peer cultural value mismatch measure loaded onto the two hypothesized typologies: (a) Factor 1 represents items related to reciprocation mismatch and (b) Factor 2 represents items related to not thinking of the other. The two-factor solution explained 63.38 % of the variance.

### Psychological distress.

A seven-item measure captured students’ feelings of distressed mood since they started UCLA. Students were asked to rate the extent (1 = *Not at all* to 5 = *Extremely*) to which they felt “on edge,” “nervous,” “uneasy,” “unable to concentrate,” “sad,” “hopeless,” and “discouraged.” This measure, previously utilized with a diverse sample (Huynh & Fuligni, 2010), is an adapted version of Lorr and McNair’s (1971) Profile of Mood States and yielded an excellent alpha of .91. The only change from the original measure was to direct participants’ attention to their time at UCLA by prefacing the instrument with “Since you started at UCLA.”

### Academic problems.

A 6-item measure of academic problems was utilized. Students were asked to rate (1 = *Never* to 5 = *Always*) how many times they experienced certain situations since they started at UCLA. These situations included attention (three-items; e.g., “had a difficult time focusing on studying”) and learning problems (three-items; e.g., “did not turn in homework that was due”; adapted from Telzer & Fuligni, 2009). The Cronbach’s alpha was .84, indicating good internal consistency.

### College GPA.

Students’ self-reports of their grade point average (GPA) earned in the fall and winter quarters of their first year at UCLA were utilized. GPAs were assessed using a scale from 4 (A) to 0 (F).

## Procedure

Participants were told that the purpose of our research was to explore home-school and peer-peer relations during the first year of college at the institution. After providing consent, participants completed an online survey via Qualtrics. The survey took students, on average 25–30 min to complete. Research participation credits or a movie ticket were provided for participation. All procedures were approved by the Institutional Review Board.

## Data Analytic Plan

The analysis concerning antecedents of peer-peer cultural value mismatch is based on the fact that, both logically and chronologically, parental education level is established long before student respondents are attending college. First-generation college students are those students whose parents had no form of postsecondary education. Therefore, first-generation status is antecedent to peer-peer cultural value mismatch. Similarly, ethnicity is established at birth, again, long before the respondent attends college. Hence, both first-generation college student status and disadvantaged minority background are intrinsically antecedent to anything that occurs during the college years, such as peer-peer cultural value mismatch. In examining antecedents of peer-peer cultural value mismatch, hierarchical linear regressions were conducted.

Correlates of peer-peer value mismatch were assessed via path analysis, a structural equation modeling technique, using Maximum Likelihood (ML) estimation in EQS 6.1 (Bentler, 2006) for Windows. A separate model for the interrelations with other variables of each type of peer-peer mismatch – reciprocation mismatch and not thinking of the other – was planned (Hypothesis 2). Separate models were appropriate to the number of observed variables (*p* = 4) and sample size (*N* = 110). A saturated model with *p* variables has *p*(*p* + 1)/2 free parameters to be estimated (Bentler, 2006). In the current study, there were *p* = 4 observed variables in this model, resulting in 10 parameters to be estimated. Bentler and Chou (1987) suggested a sample size of 5–10 participants for every free parameter, and this rule of thumb is consistent with our current sample size of 110.

The hypothesized models (Figure 1) were based on theoretical considerations and previous literature; separate models were tested for each of the peer-peer cultural value mismatch factors that emerged from our data. Model fit was evaluated using chi-square (*x*^2^), comparative fit index (CFI), and root mean square error of approximation (RMSEA). The model is a “good” fit if the *x*^2^ is not significant or near non-significance, the CFI is greater than or equal to .95, and RMSEA is less than or equal to .05 (Byrne, 2006). The model is of “moderate” fit when at least two of these are met (Byrne, 2006; Vasquez-Salgado & Chavira, 2014).

Once appropriate fit was established, direct and indirect effects were examined (Bentler, 2006). A direct effect is when one variable predicts another, and an indirect effect is when one variable predicts another variable through one or more intervening variables (Kline, 2011). In order to confirm whether an indirect effect was the main source of influence, a direct path (within the model) between those variables must not be statistically significant. If the direct path is significant, this implies that the indirect effect only explained part of the relation between one variable and another (Kline, 2011; Kohen et al., 2008). Means, standard deviations, and zero-order correlations for all variables of interest are presented in Table 2.

## Results

### Antecedents of Peer-Peer Cultural Value Mismatch

This section will focus on antecedents of peer-peer cultural value mismatch concerning reciprocation and not thinking of the other. Preliminary analyses revealed that disadvantaged minority students did not significantly differ from their other ethnic counterparts in mismatch concerning reciprocation (M_disdvantaged_ = 1.87, SD = .92; M_other_ = 1.55, SD = .67), *t*(53.53) = 1.58, *p* = .120, and not thinking of the other (M_disadvantaged_ = 1.75, SD = .76; M_other_ = 1.67, SD = .63), *t*(108) = .58, *p* = .561. Nonetheless, disadvantaged minority status was included as a control variable in our analyses. We hypothesized that first-generation college students would more likely experience peer-peer cultural value mismatch and that this relationship would hold even after controlling for disadvantaged minority background. In order to test this hypothesis, a hierarchical linear regression was conducted whereby first-generation college status was examined as a predictor of mismatch in Step 1, controlling for disadvantaged minority background in Step 2. As expected, first-generation college students reported significantly higher levels of reciprocation mismatch experiences relative to continuing-generation college students, *b* = .49, *SE* = .15, *p* = .002, even after controlling for disadvantaged minority background, *b* = .47, *SE* = .18, *p* = .010. However, first-generation college status did not relate to experiences with not thinking of the other, *p* = .707.

Together, these findings suggest that first-generation college status, above and beyond disadvantaged minority status, predicts students’ experience of peer-peer value mismatch surrounding reciprocation. Thus, experience of such mismatch can be generalized to first-generation college students from all backgrounds, not just those from disadvantaged minority backgrounds. On the other hand, the findings suggest that not thinking of the other, the other form of peer-peer cultural value mismatch, is probably typical of most first-year students, regardless of sociocultural background. This point will be further elaborated in the discussion.

### Correlates of Peer-Peer Cultural Value Mismatch

This section will focus on the correlates of peer-peer cultural value mismatch. We hypothesized that peer-peer cultural value mismatch would predict psychological distress (Figure 1). In turn, we expected that psychological distress would relate to more academic problems, and that such problems would predict lower grade point average (GPA). In addition, we did not expect a significant direct link from peer-peer cultural value mismatch to GPA (Figure 1). Instead, we expected a that peer-peer cultural value mismatch would have a significant indirect effect on GPA through the paths of the intervening variables. Below, we discuss this hypothesis with two forms of mismatch.

### Reciprocation Mismatch

**Figure 2.**
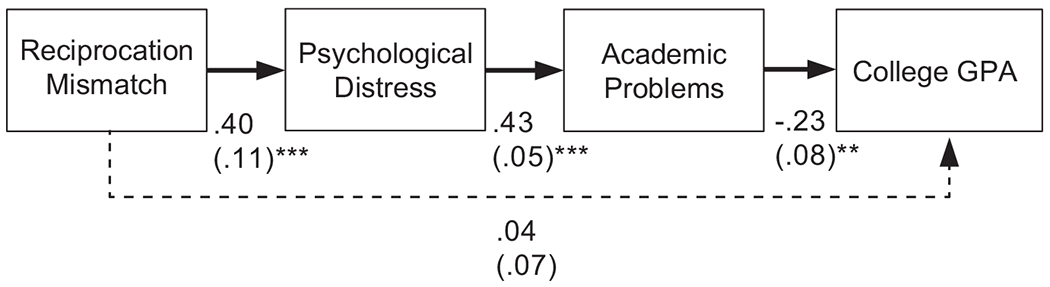
Correlates of reciprocation mismatch final model. Included are unstandardized estimates (with standard errors in parentheses); a solid line indicates significance and a dashed line indicates non-significance; *p* < .001***, *p* < .01**

This model examined relationships of reciprocation mismatch during the first year of college with psychological distress, academic problems, and GPA. As predicted, the path model (Figure 2) fit the data well, *χ*^2^(2, *N* = 110) = 1.88, *p* = .391, CFI = 1.00, RMSEA = .00. More mismatch around reciprocation predicted higher levels of psychological distress. In turn, higher levels of psychological distress predicted greater academic problems. Finally, greater academic problems related to lower college GPA during the first year.

As expected, there was no direct relation between reciprocation mismatch and GPA. Instead, there was a significant indirect effect of reciprocation mismatch on college GPA through the statistical paths of the intervening variables (i.e., reciprocation mismatch → higher levels of psychological distress → greater academic problems → lower GPA; unstandardized indirect effect = −.04, *p* = .028; standardized indirect effect = −.06), suggesting these paths as the main mechanism by which this mismatch surrounding reciprocation relates to academic performance during the first year of college.

Overall, 10 % of the variance in psychological distress, 39 % of the variance in academic problems, and 9 % of the variance in GPA was explained.

### Not Thinking About the Other

**Figure 3.**
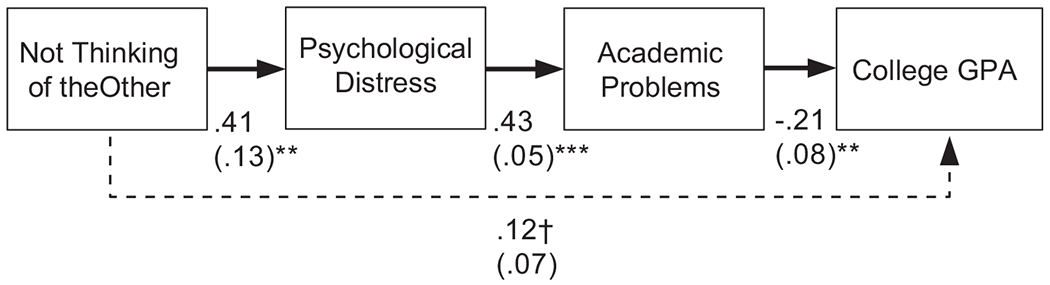
Correlates of not thinking of the other final model. Included are unstandardized estimates (with standard errors in parentheses); a solid line indicates significance and a dashed line indicates non-significance; *p* < .001***, *p* < .01**, *p* < .10†

This model examined relationships of not thinking of the other during the first year of college with psychological distress, academic problems, and GPA. Like reciprocation mismatch, the path model (Figure 3) fit the data well, *χ*^2^(2, *N* = 110) = 1.36, *p* = .505, CFI = 1.00, RMSEA = .00. As expected, more mismatch involving not thinking of the other predicted higher levels of psychological distress. In turn, higher levels of psychological distress predicted greater academic problems. Finally, greater academic problems related to lower college GPA during the first year.

In this model, there was no significant direct relation between not thinking of the other and GPA. Instead, there was also a significant indirect effect of not thinking of the other on college GPA through the statistical paths of the intervening variables (i.e., not thinking of the other → higher levels of psychological distress → greater academic problems → lower GPA; unstandardized indirect effect = −.04, *p* = .044; standardized indirect effect = −.05), suggesting these paths as the main mechanism by which this mismatch surrounding not thinking of the other relates to academic performance during the first year of college.

Overall, 8 % of the variance in psychological distress, 39 % of the variance in attention and learning problems, and 10% of the variance in GPA were explained.

## Discussion

Our findings suggest that *peer-peer cultural value mismatch* – perceived mismatch between collectivistic ideologies and practices of one student and individualistic ideologies and practices of another – in the dormitory negatively influences college adjustment during the first year of college. In addition, our findings demonstrate that first-generation college students from all backgrounds, not just disadvantaged minority students, are at risk for experiencing mismatches in reciprocation expectations.

The antecedents of *reciprocation mismatch* – giving or offering a material or a service to one’s roommate but not receiving anything in return, one type of peer-peer cultural value mismatch, was in line with expectations and prior research (Greenfield, 2009; Stephens et al., 2012a). We found that first-generation college students, regardless of disadvantaged minority background, more often experienced this form of peer-peer cultural value mismatch than continuing-generation students. This finding is in line with Cultural Mismatch Theory (Stephens et al., 2012a), which posits that first-generation college students are more likely than continuing generation students to experience a general sense of mismatch with the university culture. These findings are also aligned with the Theory of Social Change, Culture, and Human Development (Greenfield, 2009) which posits that social class, rather than ethnicity, is a main contributor to cultural values, and therefore to the experience of cultural value mismatch; in this case, being a first-generation college student was the main social-class contributor.

Contrary to our hypothesis, *not thinking of the other* – the student’s roommate was not considerate of their feelings or schedule – did not relate to either first-generation or disadvantaged minority status. This finding suggests that feeling as though your roommate is not being considerate can be due to several factors outside of cultural value mismatch and may be a situation that is more normative in roommate relations. Indeed, a review of research on roommate relationships in the dormitory noted that dormitory roommates have frequent “negotiation of responsibilities and compromises about the living environment (e.g., noise level, sleep/waking hours, visitors, and decor)” (Erb et al., 2014, p. 44). These negotiations of responsibilities and compromises about the living environment were among the items comprising our subscale pertaining to not thinking of the other (see Table 1). We therefore believe that only reciprocation is a cultural value mismatch. The other type of mismatch, not thinking of the other, may be less culture-specific and more normative to adapting to new living situations during the transition to college. Hence, to answer the secondary goal connected to our hypothesis concerning antecedents of peer-peer cultural value mismatch, only reciprocation mismatch, not lack of consideration for the other, reliably differentiates the experience of first-generation from continuing-generation students.

Nonetheless, the results confirmed our predicted model concerning the correlates of peer-peer mismatch for both reciprocation and not thinking of the other. Peer-peer mismatch in both these areas predicted psychological distress. Psychological distress, in turn, was related to a higher frequency of reported academic problems; having more problems, in turn, was associated with a lower GPA during the first year of college. More importantly, despite students’ reports that these peer-peer mismatches do not impact academic grades (Burgos-Cienfuegos et al., 2015), our quantitative results suggest otherwise – both types of mismatch (lack of reciprocation and not thinking of the other) were indirectly linked to lower grades. The indirect link between peer-peer cultural mismatch and grades provides another instance of the importance of peers in academic development, particularly the importance of roommate relations (Erb et al., 2014; Schwartz et al., 2008; Wentzel, 1998).

### Study 2

Based on the results of Study 1, Study 2 focused on the peer-peer cultural value mismatch surrounding *reciprocation* – giving or offering a material or a service to one’s roommate but not receiving anything in return (Burgos-Cienfuegos et al., 2015). We selected reciprocation mismatch because this was the peer-peer cultural value mismatch that was specific to first-generation college students in Study 1 and at the same time, was associated with negative outcomes.

However, we did not have enough information in Study 1 to determine whether this peer-peer cultural value mismatch is a function of social class differences between roommates, specifically differences in parental education. Thus, we were unable to demonstrate that students with lower levels of parental education than that of their roommate are the ones experiencing this mismatch at higher levels. We were also unable to test whether reporting more experiences with reciprocation mismatch than one’s roommate contributes to academic outcomes. Therefore, while our first study provided some support for Cultural Mismatch Theory (Stephens et al., 2012a) and the Theory of Social Change, Culture, and Human Development (Greenfield, 2009), our examination was incomplete.

The purpose of Study 2 was to survey roommate pairs. We gathered sociodemographic information from both roommates, their experience with peer-peer cultural value mismatch surrounding reciprocation, as well as their academic experiences. In so doing, we sought to carefully reexamine the antecedents and correlates of reciprocation mismatch, in a manner that takes into account responses from both roommates.

## Hypotheses

Based on the results of Study 1, as well as the aforementioned theoretical frameworks, we proposed two main hypotheses pertaining to the antecedents and correlates of mismatch concerning reciprocation. These hypotheses are detailed below.

### Antecedents of Reciprocation Mismatch (Hypothesis 1)

In line with the Theory of Social Change, Culture, and Human Development (Greenfield, 2009), we expected that there would be a significant relationship between parent education mismatch and reciprocation mismatch. Because our findings in Study 1 indicated that the lower-SES member of the roommate pair is more likely to be affected by this mismatch, we modified our hypothesis to test whether participants whose parents had lower levels of education compared with their roommate’s parents would report higher levels of reciprocation mismatch than their roommate.

This modification meant that we had to develop difference scores for each roommate individually to code not just the magnitude of educational difference between the two sets of parents but whether their parents had higher or lower education levels than their roommates. The same was true for reciprocation mismatch. Thus, our analyses could not simply use difference scores for the pair as a whole, but had to use separate difference scores for each member of the pair, as the sign of the absolute difference (positive or negative) would differ for each roommate. Details are explained in the Method section.

### Correlates of Reciprocation Mismatch (Hypothesis 2)

In line with Cultural Mismatch Theory (Stephens et al., 2012a), we predicted a significant relation between reciprocation mismatch and frequency of academic problems during the first year of college. Specifically, we hypothesized that participants who reported more experiences with reciprocation mismatch compared with their roommates (vis-a-vis the use of a difference score for each roommate) would report more academic problems.

## Method

### Participants.

At the end of an academic year at UCLA, participants were recruited via flyers posted across campus and social media, as well as through direct email. Participants had to be a freshman, in their first year of college, and live on-campus in the dormitories with the same roommate since the fall or winter quarter. In order to be invited to join the study, participants and their roommates had to complete an online prescreening. About 280 participants (140 roommate pairs) were invited to participate in our study. However, only 237 took the survey. Furthermore, because many of our variables and analyses required responses gathered from both roommates, 19 % were removed because their roommate did not complete the survey. An additional 1.6 % were removed because their roommate did not finish the survey. This process brought us to a sample size of 188.

In addition, pilot testing indicated that our survey took at least 25-minutes to complete. Minimum time needed for a given survey is an effective way to exclude careless responders (Curran, 2016). Curran further points out that careless responders are a source of noise that can potentially shift results. Individuals who spent less than 25-minutes on the survey responded in a fashion that appeared as though they were not attempting to read and answer survey questions. For example, they answered only a portion of questions or provided mostly “straight-line” responses across multiple questions; these behaviors provided further indication that fast responders were both careless and a source of noise. There is consensus in the field that 8–12 % is the modal rate of careless responding (Curran, 2016). Based on our minimum time criterion, we had 21 careless responders out of 188 participants or 11 %, so the number is within the modal range. It, therefore, seemed reasonable to eliminate participants taking less than 25 minutes in order to avoid shifting results because of noise from careless responding.

Among the 21 fast responders, six were situations where both roommates were fast responders. However, because data analysis relied on the responses of both roommates, the remaining 15 fast responders led to the elimination of 15 roommate pairs or 30 participants. This process resulted in a final sample of 152 participants (76 pairs). Sixty-four pairs identified as female; 11 pairs identified as male. In the remaining pair, one member identified as female, the other as a trans man. Ages ranged from 17 to 20 years old (*M*= 18.73, *SD* = .50). Ethnic backgrounds included Asian or Asian American (32.9 %), Latinx (27.6 %), White or European American (26.3 %), Multiracial (10.5 %), and Black or African American (2.6 %). This ethnic distribution roughly corresponded to the distribution of ethnicities at UCLA (UCLA Undergraduate Admissions, 2018–2019).

The majority of the sample was born in the United States (85 %) and none of the participants was an international or transfer student. Thus, all were first-time freshmen. Moreover, 45 % percent of participants lived with a roommate that was of the same ethnic background as themselves, and about half stated that they chose their roommate (51 %). Ninety-nine percent of the sample had lived with their roommate since Fall quarter, whereas only 1 % had lived with their roommate since the Winter quarter. Thus, almost all participants had lived with their roommates for the entire academic year – Fall, Winter, and Spring. Given that inter-group dynamics can be influenced by whether or not one’s roommate is of a similar ethnic background (Shook & Fazio, 2008a; Shook & Fazio, 2008b; Bresnahan et al., 2009) and whether or not one chooses one’s roommate (Shook & Fazio, 2008b), these variables were used as control variables in all analyses.

## Measures

### Parent education level mismatch.

Mismatch in parental education levels between dormitory roommate pairs was assessed. As an initial step, participants were asked to indicate their mother and father’s level of education. Potential education level responses ranged from (1) no formal education to (11) a professional school degree (e.g., medical, law) or doctorate degree (PhD). Thereafter, mother and father’s level of education was averaged to form one overall average of parental education for each participant.

As explained in the section on Hypothesis 1, it was necessary to calculate and use a separate difference score for each roommate, with the direction of difference indicated by a positive (if the participant’s parents had higher education than the roommate’s parents) or negative value (if the participant’s parents had lower education than the roommate’s parents). In order to determine the level of mismatch in parent education between dormitory roommate pairs, a parental education difference score was calculated for each member of a roommate pair. A participant who had parents with higher levels of education than their roommate’s parents was coded with a positive value, whereas a participant with parents who had lower levels of education than their roommate’s parents was coded with a negative value. For example, if the average level of parental education was 8 for Roommate 1 and 4 for Roommate 2, the mismatch between roommates was 4 levels of parental education. Roommate 1 would then receive a code of 4 and Roommate 2 a code of −4. Thus, this scoring system indicated level (and magnitude) as well as the direction of mismatch for each participant. In this case, the two scores would indicate a mismatch of four levels of education between the roommates and that Roommate 1’s parents were four levels higher in education than Roommate 2’s parents. In cases where there was no mismatch in education between the parents of roommate dyads, the mismatch was coded “0”. Lastly, all scores were standardized with a mean of zero and standard deviation of 1 when entered as a predictor in the regression models.

It is noteworthy to mention that this relative measure of social-class mismatch was complemented by a mean estimate of dyad-level difference in average parent education. For example, in the dyad-level mean estimate, a pair with a score of 8 and 4 are on average lower than the pair with 11 and 7, though the difference score, our relative measure, is the same 4 units. We therefore ran regression models with average parent education of both roommates as a covariate; and we found that the mismatch results remained. These findings echo our intraclass correlation coefficient (ICC) preliminary analysis (see Data Analytic section), which suggested small dyad-level effects. Given the potential multicollinearity with our variable of interest (i.e., parent education mismatch) and sample size considerations, we did not include average roommate parent education in the final models.

Although we considered techniques of dyadic analysis, such as the Actor-Partner Interdependence Model, our sample size at the pair level (*n* = 76 roommate pairs) was not sufficient for rules of thumb that call for at least 100 dyads (Kline, 2011). In addition, our hypotheses dealt with individual-level (within-individual) effects rather than the roommate-level (within-dyad) effects; and so we measured parent-education differences as they were experienced by each individual roommate.

### Reciprocation mismatch.

The 10-item measure of peer-peer cultural value mismatch described in Study 1 was administered. However, only the 5-items that assessed reciprocation mismatch were utilized (α = .76). A mean score across the five items was calculated.

In order to determine the level of misalignment in reciprocation mismatch between dormitory roommate pairs, a reciprocation mismatch difference score was calculated for each member of a roommate pair. If there was a difference, the roommate with the higher average of reciprocation mismatch was assigned a positive value and the roommate with the lower average of reciprocation mismatch was assigned a negative value. For example, if Roommate 1’s average was a 4 and Roommate 2’s average was a 2, this resulted in a difference of 2; however, Roommate 1 was assigned a 2 and Roommate 2 was assigned a −2. Thus, the difference between each roommate’s perception of mismatch was quantified as to magnitude and direction. If both roommates reported a similar level of reciprocation, the mismatch remained as “0”. Lastly, all scores were standardized with a mean of zero and standard deviation of 1 when entered as a predictor in the regression models.

### Academic problems.

The same measure of academic problems described in Study 1 was utilized (α = .81).

## Procedure

Interested participants completed an online prescreening and informed consent form and were told that they would be invited if they and their roommate completed the prescreening and were both freshmen. Participants were invited to participate by a direct email sent to both roommates. In the event that three roommates rather than two submitted prescreening responses, two were randomly selected and emailed to join the study. The email included a link to the online survey and participants were encouraged to take the survey independent of each other and not to share their responses with one another. They were also told that their survey responses would be completely confidential. Participants received $10 cash for participating in the study as well as a $10 cash bonus if they and their roommate both completed the survey. Thus, the total possible compensation for each participant was $20 cash. All procedures were approved by the UCLA Institutional Review Board.

## Data Analytic Plan

In order to test the antecedents and correlates of reciprocation mismatch, hierarchical linear regressions were modeled with mismatch (parent education level mismatch or reciprocation mismatch) variables in Step 1 and control variables (i.e., ethnic similarity, roommate selection) in Step 2.

Because participants were clustered in roommate pairs, we also calculated the intraclass correlation coefficients (ICCs) between the dyad members. In the current study, we found the ICCs were not significant (.14, −.10; *p*s = .211, .386, respectively). This suggests that small proportions of the variance were explained by the dyadic structure of the data. This result is in line with previous findings showing that most behaviors are uncorrelated in roommate pairs (Eisenberg et al., 2013). This absence of correlation confirms the decision, explained earlier, to utilize scores for each individual participant in a roommate pair. Additionally, the design effects, an indicator of how much standard errors are underestimated calculated as 1 + (*c* – 1) x ICC where *c* is the average cluster size, were less than the critical value of 2 (Peugh, 2010). Therefore, to conserve power, we tested hypotheses using single-level analyses.

## Results

### Antecedents of Reciprocation Mismatch

We hypothesized that participants with lower levels of parental education compared to their roommate would report higher levels of reciprocation mismatch than their roommate. A hierarchical linear regression was modeled to test for a direct effect. As noted earlier, parent education was considered an antecedent because it occurs prior in time to the respondents attending college.

As expected, lower levels of parental education compared to one’s roommate predicted more experiences with reciprocation mismatch than one’s roommate, *b* = −.11, *SE* = .06, *p* = .048, even after controlling for ethnic similarity and roommate choice, *b* = −.11, *SE* = .06, *p* = .049. Together, these findings imply that, in terms of antecedents of peer-peer cultural value mismatch pertaining to reciprocation, having lower levels of parental education compared to one’s roommate is linked with reporting a higher degree of mismatch about reciprocation than one’s roommate. Looked at another way, the greater the gap in parental education between the two roommates, the greater the mismatch that is experienced by the roommate whose parents had less formal education.

### Correlates of Reciprocation Mismatch

We hypothesized that participants who reported more experiences with reciprocation mismatch compared with their roommate would also report more academic problems. A hierarchical linear regression was conducted to test for a direct effect.

In line with our prediction, more experiences with mismatch about reciprocation compared to one’s roommate predicted more academic problems, *b* = .12, *SE* = .05, *p* = .017, even after controlling for ethnic similarity and roommate choice, *b* = .12, *SE* = .05, *p* = .015. Therefore, in terms of the correlates of peer-peer cultural value mismatch, these findings suggest that the greater the gap between a student’s experience of reciprocation mismatch and the mismatch experience reported by their roommate, the more academic problems the roommate experiencing mismatch will have during the transition to college.^2^

## Discussion

Our findings suggest that the antecedents and correlates of *reciprocation mismatch* – a peer-peer cultural value mismatch consisting of giving or offering a material or service to one’s roommate but not receiving anything in return – continue to hold true when reexamined with roommate pairs living together in a university dormitory. We found that social class differences in parental education between dormitory roommates played a role in students’ experiences with reciprocation mismatch: Students whose parents have less education reported significantly more mismatch than their roommates whose parents have attained a higher level of education. We also found that students who reported more mismatch than their roommates reported significantly higher levels of academic problems during the transition to college.

The greater the gap between the educational level of the two roommates’ parents, the greater the reciprocation mismatch reported by the roommate whose parents had the lower educational level. This finding, in line with our expectations, implies that, in a college dormitory, first-year students from households with significantly lower levels of parental education compared with their roommates will more often experience situations where they feel as though they offer materials or services to their roommates but do not receive anything back in return (e.g., food, academic resources, social and emotional support). This finding is directly in line with the Theory of Social Change, Culture and Human Development which suggests that transition from a social ecology characterized by collectivistic values (lower levels of parental education) to one that is more individualistic (higher levels of parental education of one’s roommates) may result in cultural value mismatch (Greenfield, 2009).

Also, as expected, the experience of cultural mismatch vis a vis reciprocation had negative implications for academic progress: The greater the reciprocation mismatch participants reported compared with their roommate, the more academic problems they reported. This finding suggests that first-year students who experience significantly more situations of reciprocation mismatch than their roommates will also experience more academic problems (e.g., difficulty with focusing, not turning in homework assignments). These findings are in line with Cultural Mismatch Theory (Stephens et al., 2012a) as well as survey and experimental work suggesting that students who experience a mismatch with the university environment are more likely to experience academic difficulties. Our findings also align with qualitative, survey and experimental work demonstrating the negative consequences of *home-school cultural value mismatch* – mismatch between collectivistic family obligations and individualistic academic obligations. This mismatch between value priorities in the home environment and value priorities in the college environment disrupts students’ ability to focus and learn in an academic environment (Vasquez-Salgado et al., 2015; Vasquez-Salgado et al., 2018; Vasquez-Salgado et al., 2021).

### General Discussion

Our research program is unique in exploring the phenomena of cultural mismatch in the context of peer relations, and the implications this mismatch has for students’ health and academic outcomes. We began with a qualitative study that explored Latinx first-generation college students’ (students whose parents had stopped their formal education at high school or less) lived experiences with peer-peer cultural value mismatch (Burgos-Cienfuegos et al., 2015). We then used survey methodology to generalize the findings to first-generation college students from other backgrounds in a larger multiethnic sample (Study 1), specifically for the experience of *reciprocation mismatch* – a mismatch consisting of giving or offering a material or service to one’s roommate but not receiving anything in return. Our survey methodology also enabled us to detail the interconnected nature concerning the health and academic costs associated with this mismatch. This interconnection is aligned with other forms of mismatch (Vasquez-Salgado et al., 2021).

By enlisting roommate pairs in Study 2, we were able to specify differences in parental education as the sociodemographic factor generating the experience of cultural mismatch. Quantifying differences in parental education, we found that these differences influenced whether or not and to what extent college roommates experienced reciprocation mismatch: Students whose parents had attained a lower education level than their roommate’s parents reported significantly more mismatch than their roommate. In addition, we also found that students who reported more mismatch than their roommate reported significantly more academic problems during the transition to college. The findings of Study 2 solidify the role of social class as a determinant of reciprocation mismatch, that is, a student feeling as though they offer materials (e.g., food) or help (e.g., emotional support) to their roommate, but do not receive anything back in return. Earlier research demonstrated that individuals of lower social class are more likely to give to others (Piff et al., 2010). The current research demonstrates that when giving is not reciprocated, reciprocation mismatch is more salient for those coming from a lower social class background. This salience negatively disrupts academics.

However, extrapolating out findings across both studies, peer-peer cultural value mismatch may be more disturbing both emotionally and academically to one member of the roommate pair: the one from the lower SES background. This is in line with earlier findings that priming individualistic values creates stress and reduces performance on academic tasks for first-generation college students, whereas priming collectivistic values creates neither stress nor reduced performance on academic tasks for continuing-generation students (Stephens et al., 2012a; Stephens et al., 2012b). We have to conclude that cultural differences are much less disturbing for those in a relatively high position in the social class hierarchy who subscribe to the dominant value system of universities. On the other hand, cultural differences are much more disturbing if one’s family occupies a relatively low position in the social-class hierarchy and one subscribes to a value system that is less accepted by higher education at large.

Our findings thus provide new instantiation of the theoretical idea that sociodemographic differences can result in cultural mismatch (Greenfield, 2009). They also extend empirical findings concerning the experience and costs of cultural mismatch among first-generation college students (Stephens et al., 2012a) to the context of peer relations.

## Limitations and Future Directions

Though the results of the current research are fruitful, they had three main limitations. The first was that both of our studies were cross-sectional in nature. In Studies 1 and 2, despite there being an intrinsic chronological sequence of antecedents (as parental education of participants and roommate dyads occurred before mismatch), this was not the case for the correlates of peer-peer cultural mismatch. As a result, though we found links between peer-peer cultural value mismatch and academic problems, the exact direction of this link cannot be confirmed; and, at the same time, it could be that a third variable, such as a general sense of interpersonal difficulties, explained these relations. Indeed, differences or “discrepancies” in perceptions of self and other can affect interpersonal relationships (e.g., Barranti et al., 2016). Longitudinal data, that include control for general interpersonal difficulties, would enable us to more rigorously ascertain how this process unfolds over time (Selig & Preacher, 2009). Our current and future research aims to address this limitation through longitudinal investigation of peer-peer cultural value mismatch in order to uncover how their relations with health and academic outcomes unfold over time. Based on the present research and cultural mismatch theory, we expect that this future research will establish that mismatch creates psychological distress, and as a result, creates academic problems that ultimately lead to lower grades over time.

Second, it is noteworthy to mention a potential limitation of Study 2. Specifically, because we required both roommates to take the prescreening in order to be invited, it is possible that we missed roommates who experienced extreme difficulties with one another. Our study required two roommates to cooperate enough for both members of the pair to take our survey. However, even in a sample without extreme roommate conflict, negative correlates of cultural mismatch within the dormitory setting were evident. Future researchers are encouraged to invite all roommates residing in a dormitory room, rather than just a dyad, in order to mitigate these selection effects.

The third limitation was that our conceptualization of peer-peer cultural mismatch was based on the qualitative experiences of one ethnocultural group – Latinx first-generation college students and encapsulated a collectivistic cultural perspective (Burgos-Cienfuegos et al., 2015). We expect that roommates from more individualistic backgrounds also experience peer-peer cultural value mismatch from their own cultural perspective; we hope to explore the ramifications of cultural mismatch from the individualistic perspective in future studies.

## Implications for Research and Practice

Based on the only pre-existing empirical study on peer-peer cultural value mismatch, qualitative in nature (Burgos-Cienfuegos et al., 2015), we have quantified the experience by developing a new measure that demonstrates construct validity. This measure of peer-peer value mismatch will enable future researchers to further test the phenomenon of reciprocation mismatch in different kinds of post-secondary institutions as well as to include other variables in order to gain an in-depth understanding of risk and resilience factors contributing to antecedents and correlates of this mismatch. Although the second form of peer-peer mismatch, not thinking of the other, is included in this measure, we have concluded that this is not a mismatch produced by cultural value differences, but rather a situation that may be a normative part of adjusting to roommates during the transition to college (Erb et al., 2014).

Our research speaks to another phenomenon on many college campuses, which is for college administers to pair incoming students of different backgrounds in the same dorm to encourage interacting across various diversity-related dimensions. While these attempts are well-intended, findings in our research suggest they may backfire. However, diversity is essential in higher education in order to prepare students to interact and engage with multiple viewpoints (Milem, 2003). In addition, extensive research with younger groups indicates that diversity exposure is vital to social development, as well as mental health and academic outcomes (Graham, 2018). Therefore, rather than focusing on unanticipated consequences, we focus implications on encouraging awareness and programmatic development in residential life communities.

That is to say, the role of social class mismatch in students’ experience with peer-peer cultural mismatch uncovers the great need for interventions that augment cross-cultural understanding among student peers in academic settings. Currently, there is one intervention available that involves panel discussion where students of different social class backgrounds discuss their difficulties with transitioning to the university (conducted in-person: Stephens et al., 2014; conducted online: Townsend et al., 2019). Perhaps a similar intervention could be conducted in residential life communities, with a focus on cross-cultural understanding between roommates.

Taken together, our two studies have provided greater understanding of antecedents and correlates of peer-peer cultural value mismatch, an understudied but important site of cultural mismatch. This is an important topic, given the ever-growing diversity and disparities that exists among social class groups in post-secondary education, as well as the general mismatch and tension among social-class groups in the United States. Many students attend college to enhance their knowledge about the world. Our research findings suggest that an important goal for instructors, staff, and mentors should be to provide students with the opportunity to enhance their knowledge about other cultures and perspectives and to provide tools necessary to help them navigate interactions between students from differing social-class backgrounds. It is particularly crucial that residential life communities across college campuses incorporate awareness, discussion, and resolution of these mismatches into their programmatic endeavors.

## Figures and Tables

**Table 1.** Factor Loadings for Items of Peer-Peer Cultural Value Mismatch

Item Number	Item	Factor 1	Factor 2
	Since you started rooming with X, please state how often have you experienced the following with him or her…		
1	I purchase items (e.g., food, snacks) that I offer to my roommate but he/she never does the same for me	**.731**	.130
2	When going somewhere (e.g., store, coffee shop), I ask my roommate if he/she wants/needs anything but he/she never does the same for me	**.902**	−.112
3	I have shared academic resources with my roommate (e.g., notes, books), but he/she has not reciprocated the same for me	**.860**	.000
4	I have shown support to my roommate when needed but he/she has not reciprocated support when I need it	**.887**	−.036
5	I often find myself helping my roommate with things she needs more than she does for me	**.784**	.039
6	My roommate uses my personal items (e.g., shampoo, food) without replacing them	.053	**.705**
7	My roommate uses my personal items (e.g., dishes) and doesn’t clean them	.285	**.518**
8	I often find myself cleaning common areas (e.g., restroom, trash) that my roommate and I both use because he/she never helps clean	.052	**.674**
9	My roommate makes a lot of noise (e.g., watches television, talks on the phone) when I am trying to study	−.022	**.820**
10	My roommate does things that disrupt my sleep (e.g., turns on lights, makes noise, brings people over)	−.163	**.875**

*Note:* Factor loadings in boldface are > .40; Factor 1 = Reciprocation mismatch; Factor 2 = Not thinking of the other; Principal components analysis with promax rotation and Kaiser normalization was conducted.

**Table 2.** Means, Standard Deviations and Zero-order Correlations for Variables of Interest in Models Assessing Correlates of Peer-Peer Cultural Value Mismatch

	1.	2.	3.	4.	5.
1. Reciprocation Mismatch	1				
2. Not Thinking of Other	.46***	1			
3. Psychological Distress	.32***	.29**	1		
4. Academic Problems	.19+	.20*	.63***	1	
5. College GPA	−.11	−.21*	−.28**	−.29**	1
Mean	1.63	1.70	2.71	2.82	3.13
Stand. Dev.	.76	.67	.96	.65	.53

*Note:*

*** *p* < .001,

** *p* < .01,

* *p* < .05,

+ p < .10

## Footnotes

1 Latinx is currently preferred in the U.S. over the terms Latino or Latina because it is not gendered; it is preferred over the term Hispanic because it does not make reference to Spain, the colonial power in this part of the world.

2 The authors also ran regression analyses using difference scores per variable in the antecedents and correlates models, reducing the data to a single row of observations per roommate pair. Single-level regression analyses of difference scores can provide a similar estimate of individual-level effects when compared with mixed-effects models with random intercepts for each cluster or dyad (Desai & Begg, 2008). The findings for the correlates model remained, *b* = .34, *SE* = .14, *p* = .021. However, the antecedents model, though trending in the expected direction, was no longer significant, *b* = .04, *SE* = .03, *p* = .137. One reason for the loss of statistical significance was the reduction in power because our sample size decreased from 152 to 76 observations. However, our analytic goal was not simply to test the effect of differences in parental education on roommate pairs, but to assess the effect of differences in parent education on that member of the pair who came from the family with lower education. Hence, the use of paired differences alone was inappropriate to establish this effect. To reach our analytic goal, it was necessary to distinguish between whether each member of the pair came from the lower or higher education background, as well as measuring the size of the educational disparity between the roommates’ families. Ultimately, the statistical analyses utilized in the main text were most appropriate in testing the hypotheses given the theoretical framing and low ICCs.

## References

[R1] American College Health Association (2019, Spring). American College Health Association – National college health assessment II: Reference group executive summary. American College Health Association.

[R2] Barranti M, Carlson EN, & Furr RM (2016). Disagreement about moral character is linked to interpersonal costs. Social Psychological and Personality Science, 7(8), 806–817. 10.1177/1948550616662127

[R3] Bentler PM (2006). EQS 6 structural equations program manual. Multivariate Software, Inc. https://www.mvsoft.com/wp-content/uploads/2021/04/EQS_6_Prog_Manual_422pp.pdf

[R4] Bentler PM, & Chou CP (1987). Practical issues in structural modeling. Sociological Methods & Research, 26(1), 78–117. 10.1177/0049124187016001004

[R5] Boettcher ML, Eason A, Earnest K, & Lewis L (2019). The cultivation of support networks by students of color in a residence hall setting at a predominantly white institution. Journal of College and University Student Housing, 45(2), 30–46. https://www.researchgate.net/publication/334748969_The_Cultivation_of_Support_Networks_by_Students_of_Color_in_a_Residence_Hall_Setting_at_a_Predom-inantly_White_Institution

[R6] Brannon TN, Markus HR, & Taylor VJ (2015). “Two souls, two thoughts,” two self-schemas: Double consciousness can have positive academic consequences for African Americans. Journal of Personality and Social Psychology, 108(4), 586–609. 10.1037/a0038992 25844575

[R7] Bresnahan MJ, Guan X, Shearman SM, & Donohue WA (2009). Roommate conflict: Does race matter?. The Howard Journal of Communications, 20(4), 394–412. 10.1080/10646170903303840

[R8] Burgos-Cienfuegos R, Vasquez-Salgado Y, Gracia-Ruedas N, & Greenfield PM (2015). Disparate cultural values and modes of conflict resolution in peer relations: The experience of Latino first-generation college students. Hispanic Journal of Behavioral Sciences, 37, 365–397. 10.1177/0739986315591343

[R9] Byrne BM (2006). Structural equation modeling with EQS: Basic concepts, applications, and programming (2nd ed.). Multivariate Applications Series. Routledge. https://psycnet.apa.org/record/2006-03173-000

[R10] Curran PG (2016). Methods for the detection of carelessly invalid responses in survey data. Journal of Experimental Social Psychology, 66, 4–19. 10.1016/j.jesp.2015.07.006

[R11] DeAngelo L, Franke R, Hurtado S, Pryor JH, & Tran S (2011). Completing college: Assessing graduation rates at four-year institutions. Los Angeles: Higher Education Research Institute, UCLA. https://heri.ucla.edu/DARCU/CompletingCollege2011.pdf

[R12] Demetriou C, Meece J, Eaker-Rich D, & Powell C (2017). The activities, roles, and relationships of successful first-generation college students. Journal of College Student Development, 58(1), 19–36. 10.1353/csd.2017.0001

[R13] Dennis JM, Phinney JS, & Chuateco LI (2005). The role of motivation, parental support, and peer support in the academic success of ethnic minority first-generation college students. Journal of College Student Development, 46(3), 223–236. 10.1353/csd.2005.0023

[R14] Desai M, & Begg MD (2008). A comparison of regression approaches for analyzing clustered data. American Journal of Public Health, 98(8 ), 1425–1429. 10.2105/AJPH.2006.108233 18556621 PMC2446458

[R15] Eisenberg D, Goberstein E, & Whitlock JL (2014). Peer effects on risky behaviors: New evidence from college roommate assignments. Journal of Health Economics, 33, 126–138. 10.1016/j.jhealeco.2013.11.006 24316458

[R16] Erb SE, Renshaw KD, Short JL, & Pollard JW (2014). The importance of college roommate relationships: A review and systemic conceptualization. Journal of Student Affairs Research and Practice, 51(1), 43–55. 10.1515/jsarp-2014-0004

[R17] Fuligni AJ, & Pedersen S (2002). Family obligation and the transition to young adulthood. Developmental Psychology, 38(5), 856–868. 10.1037/0012-1649.38.5.856 12220060

[R18] Gan S, Cheah M, Chen K, & Wong L (2019). The effects of parental and peer attachment on university adjustment among first-year undergraduate atudents. Advances in Social Science, Education and Humanities Research, 229, 682–691. 10.2991/iciap-18.2019.58

[R19] Graham S (2018). Race/ethnicity and social adjustment of adolescents: How (not if) school diversity matters. Educational Psychologist, 53, 64–77. 10.1080/00461520.2018.1428805

[R20] Greenfield PM (2009). Linking social change and developmental change: Shifting pathways of human development. Developmental Psychology, 45, 401–418. 10.1037/a0014726 19271827

[R21] Greenfield PM, Keller H, Fuligni A, & Maynard A (2003). Cultural pathways through universal development. Annual Review of Psychology, 54(1), 461–490. 10.1146/annurev.psych.54.101601.145221 12415076

[R22] Greenfield PM, & Quiroz B (2013). Context and culture in the socialization and development of personal achievement values: Comparing Latino immigrant families, European American families, and elementary school teachers. Journal of Applied Developmental Psychology, 34, 108–118. 10.1016/j.appdev.2012.11.002

[R23] Huynh VM, and Fuligni AJ (2010). Discrimination hurts: The academic, psychological, and physical well-being of adolescents. Journal of Research on Adolescence, 20, 916–941. 10.1111/j.1532-7795.2010.00670.x

[R24] Jaggers D, & Iverson SV (2012). “Are you as hard as 50 cent?” Negotiating race and masculinity in the residence halls. Journal of College and University Student Housing, 38/39(2/1), 186–199. https://eric.ed.gov/?id=EJ1014031

[R25] Jenkins SR, Belanger A, Connally ML, Boals A, & Durón KM (2013). First-generation undergraduate students’ social support, depression, and life satisfaction. Journal of College Counseling., 16(2), 129–142. 10.1002/j.2161-1882.2013.00032.x

[R26] Juang L, Ittel A, Hoferichter F, & Miriam Gallarin M (2016). Perceived racial/ethnic discrimination and adjustment among ethnically diverse college students: Family and peer support as protective factors. Journal of College Student Development, 57(4), 380–394. 10.1353/csd.2016.0048

[R27] Kline R (2011). Principles and practices of structural equation modeling (3rd ed). Guilford Press. https://psycnet.apa.org/record/2010-18801-000

[R28] Kline RB (2005). Principles and practice of structural equation modeling (2nd ed.). Guilford.

[R29] Kohen DE, Leventhal T, Dahinten VS, & McIntosh CN (2008). Neighborhood disadvantage: Pathways of effects for young children. Child Development, 79, 156–169. 10.1111/j.1467-8624.2007.01117.x 18269515

[R30] Lightfoot C, Cole M, & Cole SR (2018). The development of children (8th ed.). Worth Publishers.

[R31] Liston C, McEwen BS, & Casey BJ (2008). Psychosocial stress reversibly disrupts prefrontal processing and attentional control. Proceedings of the National Academy of Sciences, 106, 912–917. 10.1073/pnas.0807041106 PMC2621252 19139412

[R32] Liu A, Sharkness J, & Pryor JH (2008). Findings from the 2007 administration of your first college year (YFCY): National aggregates. Los Angeles, CA: Higher Education Research, UCLA. https://www.heri.ucla.edu/PDFs/YFCY_2007_Report05-07-08.pdf

[R33] Lorr M, & McNair DM (1971). The profile of mood states manual. Educational Industrial Testing Service.

[R34] Martin MM, & Anderson CM (1995). Roommate similarity: Are roommates who are similar in their communication traits more satisfied? Communication Research Reports, 12, 46–52. 10.1080/08824099509362038

[R35] Mathews A, & MacLeod C (1985). Selective processing of threat cues in anxiety states. Behaviour Research and Therapy, 23, 563–569. 10.1016/0005-7967(85)90104-4 4051929

[R36] Maunder RE (2018). Students’ peer relationships and their contribution to university adjustment: the need to belong in the university community. Journal of Further and Higher Education, 42(6), 756–768. 10.1080/0309877x.2017.1311996

[R37] Meyers JE, Grills CE, Zellinger MM, & Miller RM (2014). Emotional distress affects attention and concentration: The difference between mountains and valleys. Applied Neuropsychology: Adult, 21, 28–35. 10.1080/09084282.2012.721148 24826493

[R38] Milem JF (2003). The educational benefits of diversity: Evidence from multiple sectors. In Chang MJ, Witt D, Jones J, & Hakuta K (Eds.), Compelling interest: Examining the evidence on racial dynamics in higher education (pp. 126–169). Stanford University Center for Comparative Studies in Race and Ethnicity.

[R39] Newton RR & Rudestam KE (1999). Your statistical consultant: Answers to your data analysis questions. Sage.

[R40] Peugh JL (2010). A practical guide to multilevel modeling. Journal of School Psychology, 48(1), 85–112. 10.1016/j.jsp.2009.09.002 20006989

[R41] Phillips LT, Stephens NM, Townsend SS, Goudeau S (2020). Access is not enough: Cultural mismatch persist to limit first-generation students’ opportunities for achievement throughout college. Journal of Personality and Social Psychology, 119, 1112–1131. 10.1037/pspi0000234 32105102

[R42] Piff PK, Kraus MW, Côté S, Cheng BH, & Keltner D (2010). Having less, giving more: The influence of social class on prosocial behavior. Journal of Personality and Social Psychology, 99(5), 771–784. 10.1037/a0020092 20649364

[R43] Posselt JR, & Lipson SK (2016). Competition, anxiety, and depression in the college classroom: Variations by student identity and field of study. Journal of College Student Development, 57(8), 973–989. 10.1353/csd.2016.0094

[R44] Ramirez G, & Beilock SL (2011). Writing about testing worries boosts exam performance in the classroom. Science, 333, 211–213. 10.1126/science.1199427 21233387

[R45] Rozek CS, Ramirez G, Fine RD, & Beilock SL (2019) Reducing socioeconomic disparities in the STEM pipeline through student emotion regulation. Proceedings of the National Academy of Sciences, 5, 1553–1558. 10.1073/pnas.1808589116 PMC6358706 30642965

[R46] Sadoughi M, & Hesampour F (2016). Relationship between social support and loneliness and academic adjustment among university students. International Journal of Academic Research in Psychology, 3(2), 1–8. 10.6007/ijarp/v3-i2/2455

[R47] Saenz VB, Hurtado S, Barrera D, Wolf D, & Yeung F (2007). First in my family: A profile of first-generation college students at four-year institutions since 1971. Los Angeles, CA: Higher Education Research Institute. https://www.heri.ucla.edu/PDFs/pubs/TFS/Special/Monographs/FirstInMyFamily.pdf

[R48] Schwartz D, Hopmeyer Gorman A, Dodge KA, Pettit GS, & Bates JE (2008). Friendships with peers who are low or high in aggression as moderators of the link between peer victimization and declines in academic functioning. Journal of Abnormal Child Psychology, 36, 719–730. 10.1007/s10802-007-9200-x 18330690 PMC2760378

[R49] Selig JP, & Preacher KJ (2009). Mediation models for longitudinal data in developmental research. Research in Human Development, 6, 144–164. 10.1080/15427600902911247

[R50] Shook NJ, & Fazio RH (2008a). Interracial roommate relationships: An experimental field test of the contact hypothesis. Psychological Science, 19(7), 717–723. 10.1111/j.1467-9280.2008.02147.x 18727788

[R51] Shook NJ, & Fazio RH (2008b). Roommate relationships: A comparison of interracial and same-race living situations. Group Processes & Intergroup Relations, 11(4), 425–437. 10.1177/1368430208095398

[R52] Sriram R, Haynes C, Weintraub SD, Cheatle J, Marquart CP, & Murray JL (2020). Student demographics and experiences of deeper life interactions within residential learning communities. Learning Communities Research and Practice, 8(1), Article 8. https://eric.ed.gov/?id=EJ1251603

[R53] Stephens NM, Fryberg SA, Markus HR, Johnson CS, & Covarrubias R (2012a). Unseen disadvantage: how American universities’ focus on independence undermines the academic performance of first-generation college students. Journal of Personality and Social Psychology, 102, 1178–1197. 10.1037/a0027143 22390227

[R54] Stephens NM, Hamedani MG, & Destin M (2014). Closing the social-class achievement gap: A difference-education intervention improves first-generation students’ academic performance and all students’ college transition. Psychological Science, 25, 943–953. 10.1177/0956797613518349 24553359

[R55] Stephens NM, Townsend SSM, Markus HR, & Phillips LT (2012b). A cultural mismatch: Independent cultural norms produce greater increases in cortisol and more negative emotions among first-generation college students. Journal of Experimental Social Psychology, 48, 1389–1393. 10.1016/j.jesp.2012.07.008

[R56] Suárez-Orozco C, & Suárez-Orozco MM (1995). Transformations: Immigration, family life, and achievement motivation among Latino adolescents. Stanford University Press.

[R57] Telzer EH, & Fuligni AJ (2009). Daily family assistance and the psychological well-being of adolescents from Latin American, Asian, and European backgrounds. Developmental Psychology, 45(4), 1177–1189. 10.1037/a0014728 19586187

[R58] Toutkoushian RK, May-Trifiletti JA, & Clayton AB (2019). From “first in family” to “first to finish”: Does college graduation vary by how first-generation college status is defined? Educational Policy, 35(3). 10.1177/0895904818823753

[R59] Townsend SSM, Stephens NM, Smallets S, & Hamedani MYG (2019). Empowerment through difference: An online difference-education intervention closes the social class achievement gap. Personality and Social Psychology Bulletin, 45(7), 1068–1083. 10.1177/0146167218804548 30404569

[R60] Trumbull E, Rothstein-Fisch C, Greenfield PM, & Quiroz B (2001). Bridging cultures between home and school: A guide for teachers. Mahwah, NJ: Lawrence Erlbaum Associates and San Francisco: WestEd. https://www.wested.org/resources/bridging-cultures-between-home-and-school-a-guide-for-teachers/#

[R61] UCLA Undergraduate Admission (2018). UCLA Student Profile. https://admission.ucla.edu/apply/freshman/freshman-profile/2018

[R62] U.S. Department of Education, National Center for Education Statistics (2018, February). First-generation college students: College access, persistence and post-bachelor’s outcomes. https://nces.ed.gov/pubs2018/2018421.pdf

[R63] Vasquez-Salgado Y, Camacho TC, López I, Chavira G, Saetermoe CL, & Khachikian C (2023). “I definitely feel like a scientist”: Exploring science identity trajectories among Latinx students in a critical race theory-informed undergraduate research experience. Infant and Child Development, e2371. 10.1002/icd.2371 PMC12520603 41098243

[R64] Vasquez-Salgado Y, & Chavira G (2014). The transition from middle school to high school as a developmental process among Latino youth. Hispanic Journal of Behavioral Sciences, 36, 79–94. 10.1177/0739986313513718 PMC4155758 25202166

[R65] Vasquez-Salgado Y, Greenfield PM, & Burgos-Cienfuegos R (2015). Exploring home-school value conflicts: Implications for academic achievement and well-being among Latino first-generation college students. Journal of Adolescent Research, 30(3), 1–35. 10.1177/0743558414561297

[R66] Vasquez-Salgado Y, Greenfield PM, & Guan SA (2021). Home-school cultural value mismatch: Antecedents and consequences in a multi-ethnic sample transitioning to college. Frontiers in Psychology, 12. 10.3389/fpsyg.2021.618479 PMC8450350 34552520

[R67] Vasquez-Salgado Y, Ramirez G, & Greenfield PM (2018). The impact of home-school cultural value conflicts and President Trump on Latina/o first-generation college students’ attentional control. International Journal of Psychology, 53, 81–90. 10.1002/ijop.12502 29926910

[R68] Wentzel KR (1998). Social relationships and motivation in middle school: The role of parents, teachers, and peers. Educational Psychology, 90, 202–209. 10.1037/0022-0663.90.2.202

[R69] Yiend J (2010). The effects of emotion on attention: A review of attentional processing of emotional information. Cognition and Emotion, 24, 3–47. 10.1080/02699930903205698

